# Netographic narratives of user-generated travelogues on tourist destination image of Thailand

**DOI:** 10.1371/journal.pone.0301582

**Published:** 2024-05-08

**Authors:** Jinsheng (Jason) Zhu, Ling Shan

**Affiliations:** 1 Faculty of International Education and Cooperation, Guilin Tourism University, Guilin, Guangxi, China; 2 Faculty of Social Sciences, Visiting Scholar, Chiang Mai University, Chiang Mai, Thailand; 3 School of Journalism and Communication, Shanghai University, Shanghai, 200444, China; Federal University of Goias: Universidade Federal de Goias, BRAZIL

## Abstract

The image of a tourist destination is considered a vital aspect of international travel experiences, yet research in this area remains limited. Adopting a combination of netography and qualitative research methodology, this study aims to contribute to the scientific knowledge of destination image development in Thailand by analysing online travelogues to evaluate how Chinese tourists interpret the idea of destination image. To achieve this goal, 146,641 words of Chinese internet comments containing the keyword "Thailand" from four major media sources and Chinese bloggers were gathered and analysed using netography methodology. The findings showed that there was a rise in public interest, in public forums, in the destination image of Thailand among Chinese outbound tourists. The study’s results may provide important fundamental theoretical insights and inspire further investigation into the issue of destination image construction.

## Introduction

There is a significant opportunity for the travel and tourism sector to improve its market competitiveness by integrating new digital social media with more conventional marketing strategies [1–4]. New travelogue platforms have emerged as essential marketing channels to build a tourism destination [5,6], thus increasing its market competitiveness and gaining a deep description to consumers (tourists). In previous studies, academics could refer to travelogues as information sharing platforms [7,8], knowledge sharing behaviour studies [9,10], online travel reviews [11,12], etc. In addition, they are new means of communicating with audiences from all over the world and users of the Internet. Some academics have observed and researched the connection between travelogues and photographic depictions of destinations [13,14], focusing on the tourism travelogue analysis and its correlation to the tourism destination image [15,16].

Between 2018 and 2019, the influx of tourists from mainland China to Thailand reached approximately 11 million, marking a noteworthy year-over-year increase of 7.44%. Remarkably, these Chinese tourists constituted 27.53% of the total international visitors to Thailand during this period [17,18]. Despite the extensive impacts of COVID-19 pandemic, China has remained as a significant market within the Thai tourism industry [19,20]. In essence, it becomes evident that mainland China represents a substantial contributor and primary source market for Thailand’s overarching tourism economic growth and the expansion of its tourism sector, particularly in the post-pandemic era. Despite the considerable significance of the Chinese outbound market and some recent and outstanding researches [21], the genuine perspectives of Chinese tourists venturing beyond China’s borders have yet to receive adequate attention within the academic sphere and remain enigmatic. Nontheless, the interpretation of such data is beset with challenges, primarily due to the nature of tourist destination image. Firstly, a predominant portion of online assessments takes a qualitative approach, rendering it intricate to quantitatively measure and analyse the data. Secondly, the sheer volume and lack of structure in the data present difficulties in comprehending the information. Thirdly, despite the profound significance of the Chinese outbound market, a notable hindrance lies in the fact that travel accounts and social media platforms originating in China are predominantly composed in the Chinese language. Hence, it becomes paramount within the current academic landscape to gain a scholarly understanding of authentic Chinese travelogues. This understanding is critical for interpreting the viewpoints of Chinese tourists who have explored destinations outside of China, particularly in the context of Thailand. Therefore, in order to fill this current research gap, the primary aim is to utilize the considerable collection of visitor-generated content accessible on digital platforms to assist destination image agencies in understanding how travelers perceive the image of a particular destination.

To address the abovementioned research gap, this study employs a netography methodology to analyse written travelogues of Chinese tourists sourced from four major media and blog platforms. It delves into their expectations, sentiments, interpretations, and experiences during their visits to Thailand. The integration of quantitative and qualitative research methodologies enhances the study’s credibility and rigor. The main aim of this study is to identify the prominent attributes that have a substantial impact on the perception of Thailand as a tourist destination. This will be achieved by using the abundant resources available in online Chinese travelogues. The research endeavours to address the following key questions: How do individual Chinese outbound travellers perceive Thailand, one of the most popular tourist destinations? What are the self-positioning attitudes and evolving trends among new generation of Chinese outbound tourists? To what extent does this reflect a concept of modernity within the context of Chinese outbound tourists’ interactions with Thailand as a destination? In pursuit of answers to these inquiries, this study seeks to gain a comprehensive understanding of the diverse perspectives of Chinese outbound tourists by analysing travelogues sourced from prominent platforms where they share their travel experiences.

The paper established the following structural disposition: It begins with a review of prior work on tourist destination image and travelogue theories. The conceptualization to adopt netography is then described in detail, along with the coding procedure and conceptualization extraction. The perspectives of Chinese outbound tourists as depicted in the travelogues represent a changing trend in the current historical environment, consisting of imaginary, projected, and induced tourist destination images. The conclusion consists of further recommended and discursive thoughts and a conclusion section.

## Literature review

### The tourist destination image construction through travelogues

The construction of a tourism destination’s image is a complicated, methodical undertaking involving several aspects, including the destination’s attributes, social interests and values, local culture, local residents’ personality, and tourism resources [22–25]. There has been a change in the marketing function of tourism planning, where it has widened to encompass products and attractions’ innovation and business interactions. This change came about as a result of a transformation that occurred in the marketing position [22]. Using a certain categorization of destination regions and stakeholders, it is feasible to recognize the many photographic representations and social constructions of the tourist online destination image contained such classification [26]. In the process of establishing an online image of a tourist destination, the destination, in conjunction with online tourism marketers, plays an increasingly major role [27–29]. Lian [30] collects written and visual information on one tourist destination of Huangshan via the main website and through online travel advertisers, to investigate the dynamic effects of photographic and social media content to the destination image construction. Additionally, using Kenya as a case study, Muhoho-Minni’s research [31] sought to determine the role of social media in the formation of destination attributes and whether various types of media exhibit various effects on destination images and tourists’ travel choices. Through a critical examination of the tourist discourse, Natalia et al. [32] examine the subject of destination marketing and strategic communications. Jeong and Kim [33] identified the event quality of grand sporting events as a crucial factor in establishing a destination’s image.

The construction of an image is synonymous with improving the connotation of the destination image and establishing an emotional connection with the traveling customers [33,34]. From this vantage point, a fictitious image has the following degrees of concepts: popularity, reputation, propensity, association, satisfaction, and loyalty [35,36]. In a similar vein, the starting point of the current study is to comprehend the processive understanding of the Chinese outbound tourists towards the image of Thailand, as well as the inner logic of how the Chinese outbound tourists perceive the Thai destination image, by analysing their existing travelogues.

### The importance of understanding and interpreting travelogues

The study of Internet public opinion, tourism destination image, tourist satisfaction, destination reputation, and tourism population flow has made major advances in sociology in recent years [37–39]. The existing literatures have noted that the Chinese outbound tourism has witnessed fundamental transformation with the emergence of Chinese travelogues, especially to those independent travellers from China. Travelogues are also viewed as a communication platform for sources of mouth-to-mouth reputation of destinations [40,41]. Scholars argue that travelogues have the potential to be an indirect strategy for the distribution channel of destination tourism items, a new paradigm for the market place, and a method to enhance tourists’ impressions of the location [42–44]. The government agencies may employ the user generated travelogues as a marketing technique for the purpose of promoting the reputation of tourist destinations [45]. Furthermore, travelogues may serve as an instigating element that generates the desire to travel [46,47]. Participation in fam trips participated by overseas bloggers allows for the production of professional travelogues, which in turn increases the exposure of the place as a tourist destination [48–50]. Researchers on travelogue issues focus mainly on several aspects: user-generated travelogues [51], tourism enterprises’ soft advertising of tourism enterprises embedded in commercial route debriefing [52,53], travelogues generated by professional travel bloggers [48] or agencies from the governments of tourist destinations [54,55]. Chinese travelogues and social media platforms have completely transformed both the demand side and the supply side. This is due to the fact that most independent Chinese travellers read these travelogues and digital applications to help them plan their own international travels [56]. The transition on the demand side involves the growing modernity-tinged consumerism in China, the process by which Chinese tourists choose their vacation destinations, and its influential influence on the behaviour of Chinese consumers [57]. The changes in the supply side consist of the construction and promotion of tourist destination image, the marketing of tourist destination and the management of local hospitality services [58], catering for the China Ready campaign [59,60]. Some existing literatures have been concerned on tourism supply side, examining the travelogue impact on destination management and promotion [61], business-oriented marketing [62,63], as well as tourism enterprise marketing strategy and tourism products publicity [64,65].

Research on Chinese travelogues has demonstrated a notable rise in various aspects, such as the quantity of Chinese literature worldwide [66], cultural interpretations, and intercultural exchange [67]. Ma et al. [68] conducted an investigation into the aftermath feedback concerns made by Chinese tourists about the tourist destination. After the shipwreck disaster that occurred in the Phuket Islands in Thailand in 2012, Chinese bloggers also widely commented on the performance of the Thai government and thus generated an atmosphere of online public tourism crisis [69]. Nonetheless, research on how Chinese travelogues influence Chinese travellers’ own desire to travel is scarce, especially regarding Thailand. Thus, the goal of the present research is to examine Thai-themed Chinese outbound travelogues to get a more in-depth understanding to further the academic understanding of tourist destination image theories and the practical ramifications.

## Research methodology

### Methodology adopted

Netography is a research methodology that aims to provide a more comprehensive understanding of travel experiences and is frequently integrated into tourism branding strategies [70,71]. This approach considers services as the supportive process that ultimately creates value for tourists. Netography has proven to be an effective tool for investigating online spaces that reveal valuable information about customers’ value creation processes, offering unprecedented opportunities for tourism and hospitality service research. Researchers have utilized netography to investigate a broad range of topics, including consumer experiences, behaviours, and preferences, brand formation, experience consumption, consumer identity, pro-consumption, brand imaging, destination authenticity, influencers, value co-creation, and online-word of mouth.

The application of netography in the field of service management and marketing is progressively growing, and scholars believe that it has numerous promising applications. According to Heinonen and Medberg [72], netographic research offers service researchers unparalleled access to naturalistic online data, which is essential for developing a deeper understanding of service theory and practice. In the meantime, netography research approach is also an effective research method to approach to online comments in communication studies [73]. For instance, Chang et al. [74] studied the communicative blame in online communication of the pandemic using internet sources, in which they called the research methodology as “Automated Content Analysis” (see the title of the paper). These articles seek to explore how netography might enhance communication theory and practice in a digital environment with various beneficial approaches [75]. While the primary focus of this article is on netography, complementary methods and technologies, such as mobile ethnography, online interviews, immersive technologies, and the metaverse, will also be discussed.

The use of netography to Chinese travellers’ accounts of their experiences in Thailand as a destination is one of the approaches utilized in this study. Since the 1990s, netography has been used in the field of research pertaining to tourism [76–78]. Within a short period of time following that, eminent netography scientists started to emerge in the field of tourism studies. Netography places an emphasis on the reproducibility of the research process and the traceability of the research results [79]. An examination of tourist concerns is a notable approach that incorporates multiple methods in tourism research, netography, and online source research. This strategy combines netography with online source research [47,61,80–83]. In the social sciences, particularly in the tourism sector, social networking analysis is often used to decipher the latent implications of travelogues and to extract conceptions from a vast amount of substantial materials [84]. These achievements are not limited to quantitative research on manifest contents but are also applied to qualitative approaches, using text coding to investigate those implicit meanings behind the coded collected contents. A qualitative angle is turned out to be inclusive and diversified, showing that the netography approach has a good applicability and explanatory power to investigate complex issues in tourism sector that are difficult to approach. This paper adopted the netography methodology to approach both the explicit and implicit contents, which means that the paper looked at both the tangible words related to contents analysis or contained in the social networks.

In addition, this research is based on the analysis of social networks, that is, in user-generated Chinese material on the topic of the imagined image that Chinese tourists have of Thailand, as well as the shared experiences of Chinese travellers who have already been to Thailand. The media resources include of instantaneous social media, trip blogs posted online, and online news articles. The technique of netography has evolved into a methodology for the processing of information in social sciences. These Chinese travelogues are a complicated social phenomenon that entail the regular contact between Chinese outbound tourists and the hosts in a fixed location. They are written about people’s experiences traveling outside of China to Thailand.

### Text mining through selecting research source

The travelogues written by Chinese tourists who travelled to Thailand serve as a study object for this article. In particular, the emphasis of this study is on the travelogues of Chinese tourists who visited Thailand, which were collected from several of the most popular public forums and websites between January 2015 and December 2022. Travelogue data collections from the consumer side were done through the following channels through Zhihu.com (an online question and answer website similar to Quora), Xiaohongshu.com (a smartphone application that focuses on the sharing of lifestyles), Mafengwo.com (a travel blog produced by young Chinese Internet users to aid with trip planning), Tianya Club (one of China’s largest prominent Web forums and recently shut down in 2023 for unsustainable revenue generation), as well as Chinese tourists’ simultaneous messenger WeChat (a massively major social media chatting application in the world), including but not restricted to the aforementioned sources and information channels. The rationale behind utilizing a combination of netography and content analysis was motivated by the objective of achieving an overarching comprehension of the online conversation and attitudes expressed by Chinese outbound tourists. Netography facilitated our immersion in the digital networks where these travellers exchange their experiences and perspectives, so yielding valuable qualitative insights. In contrast, the utilization of content analysis allowed for the systematic quantification and analysis of the textual data obtained from these sources, so ensuring a methodical and organized approach to our investigation. The integration of these two approaches was undertaken with the objective of encompassing the rich qualitative aspects as well as the extensive quantitative aspects of our data, so providing a more holistic understanding of the Thai tourist destination image among the Chinese outbound tourists. The combined method is considered suitable, credible, and adequate to address the complex and perpetually evolving characteristics of online journey narratives.

Most of these travelogues chronicle first-hand accounts of Chinese tourists who travelled outside of China and visited Thailand. For this investigation, travelogues may provide comprehensive methodological hints and specific observational details. The Chinese travelogues collected from Chinese online platforms were then submitted to machine translation using the Deepl translation website to convert into English. The main reason for this is the vast quantity of more than 146,641 Chinese characters makes manual translation impractical. Travelogues serve as a crucial source of information in the examination of the determinants of the influx of outbound visitors from China to Bangkok and Chiang Mai cities in Thailand. Given their status as prominent tourist sites for Chinese travelers and their status as the biggest cities in Thailand, these two cities have significant importance. Through the lens of the methodology of netography, these contents are open to scrutiny using rationality and logical reasoning. The gathered data were duplicated and consolidated into a single comprehensive document before being submitted for more investigation. The aforementioned words were consolidated into a unified document with distinct paragraphs, thereafter imported into word processing software for the purpose of classification using encoding procedures. Subsequently, a definitive theoretical abstraction and a sensible generalization have been achieved. The succeeding set of conclusions will center on the philosophical and practical consequences of the results and subsequent arguments.

## Results

Sample selection is used to determine the content to be analysed. Since this paper needs to examine the issue of Thailand’s tourism destination image within the scope of Chinese tourists’ travelogues, the selection of samples also needs to take the real and effective travelogues of the research object as the core data of netography. For specific sampling methods, we refer to the guidance of the scholar Krippendorff [84] and choose the following three kinds: a) Intentional sampling. The authors conduct active screening of a wide number of text sources to identify those with the most relevant, influential, and informative material for research topics [85]. b) snowball sampling. The authors take Thailand tourism as the key words, search the travelogues of Thailand as the sample clue, and collect and supplement the contents of "Thailand tourism", "Thai people", "Thailand diet" mentioned in them at any time. In the headline of each travelogue, there must include the key words *Thailand / touring Thailand / outbound tourism / Chinese outbound tourists*, *etc*. c) Correlation sampling. After preliminary extraction of information and coding of the samples, the authors perform two targeted sampling rounds based on the amount of effective information provided by the various samples. These travelogues are intercepted and screened according to the following principles: a) travelogues with only photos or videos are excluded; b) remove travelogues with only scenic spot introduction, route recommendation, and specialty promotion; c) remove travelogues with obvious commercial contents; d) remove incomplete travelogues; e) remove repeated travelogues of the same name author from different websites. After deleting all images, videos, and other digital symbols from these documents, the authors sort the text version of 146641 Chinese characters pertaining to Thailand travelogues by Chinese outbound tourists.

**Fig 1:**
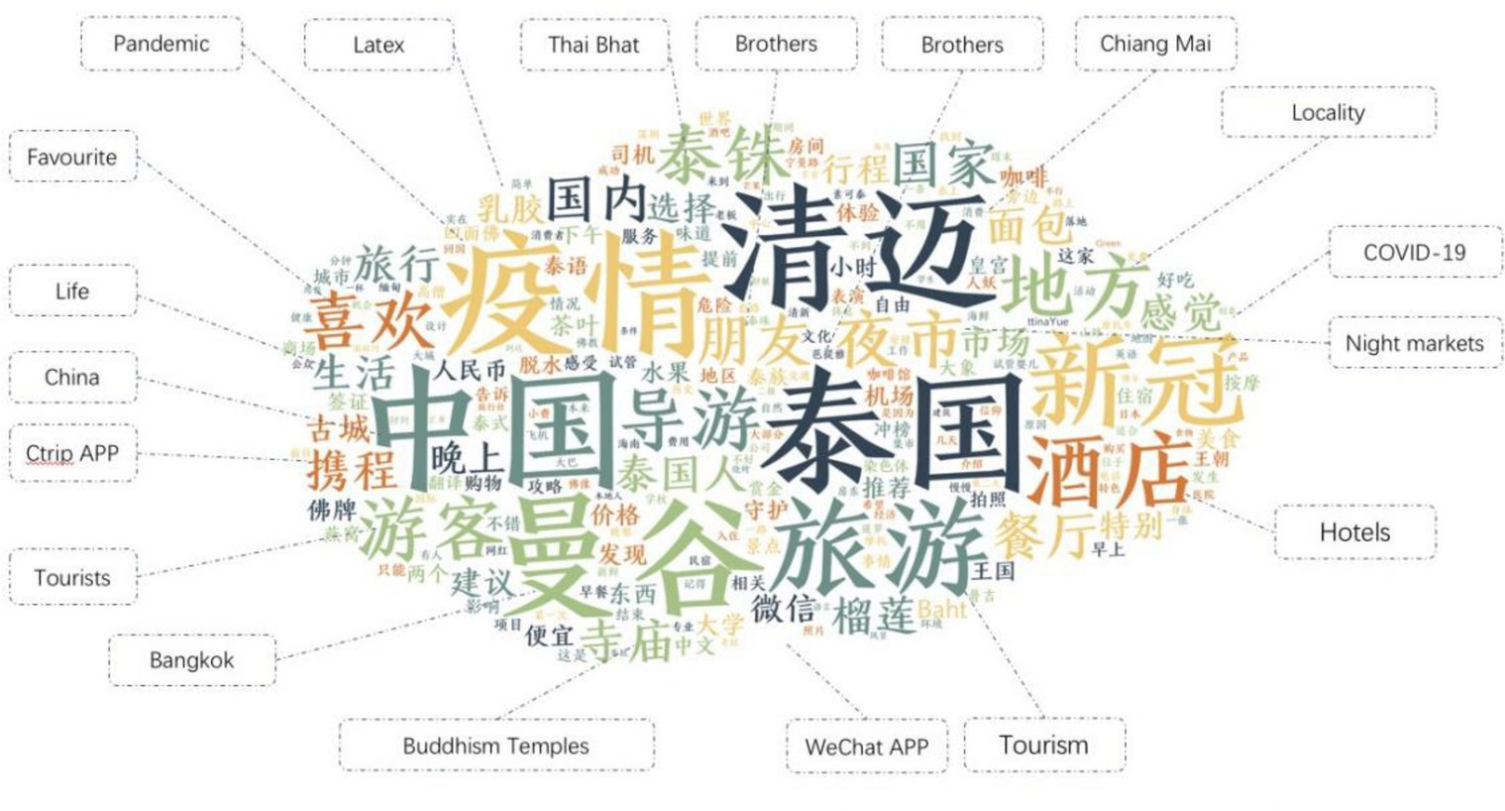
Word Cloud of the most frequently recurring keywords in Chinese travellers’ travelogues during the previous decade. (Note: The authors used word processing software for data collecting and Word Cloud graph making and analysis. The authors translated some of the words seen in the above Word Cloud diagram. This was done to facilitate English reading and further interpretation).

The study findings are shown as a graphical representation of textual information supplied in Chinese characters using a Word Cloud graph (refer to Fig 1 below). As unprocessed textual information expands at an unprecedented rate, Word Cloud enables us to examine the massive amounts of material generated. Finally, the text part of the selected travelogues is then processed and shown as follows, part of which has been translated into English words and the word frequency of the text content is then subjected to further analysis.

In addition, the authors have conducted a more in-depth investigation of the encoded and classified data. Following the conclusion of the classification and summarization procedures, the original texts of the sentences are updated, and software is then used to extract major categories with the greatest frequency for further assessment(please refer to Table 1 below). These information codes are basically a compilation of the tourist experiences and comments provided by Chinese tourists who visited Thailand. The collected keywords likely contribute to Thailand’s reputation as a global tourism destination for Chinese tourists traveling internationally.

**Table 1:** Netography value criteria for coding through frequently mentioned keywords by Chinese outbound tourists in travelogues.

Coding category	Coding criteria	Coding contents
Text Information	The total text volume should reach the level of credibility and reliability	146641 Chinese characters are sufficient for analyzing the authentic perceptions of the Chinese outbound tourists
Value for coding	Relevance of the content with the issue of Thailand’s tourist destination image	Locality, Culture, Kingdom, Temple, Hotel, Restaurants, Coffee, Airport. . .
	Interconnect to the pre-tour imaginations	Travelling Guidelines, Local Friends, Landscape, Sea sides, Freedom, Cozy, Characteristics, Friendly, Healthy, Buddhism……
	Related to the onsite travel experiences	Thai Bhat, Motorbike, Shopping, Purchasing, Consuming, Prices, Home Stay, Impressive, Feelings, The Kingdom…
	Reflect on the post-tour impression	Recommendation to friends, Word-of-mouth impressions, Local Specialty, Refreshing, Peace, Tourist Souvenirs……

As noted previously, after the coding and sorting of sample data has been finished, the authors utilize the coding coverage statistics tool to analyse the use of various kinds of samples. It also indicates that the original sample collection was substantially saturated with the material collected from these four sources. The specific method is to collect part of the tourism experience with key words of specific incidents. Part of the analysis and discussion below will focus on some specific topics coded based on the issues of tourists’ imaginary, travel experiences, and post-tour impressions.

**Fig 2:**
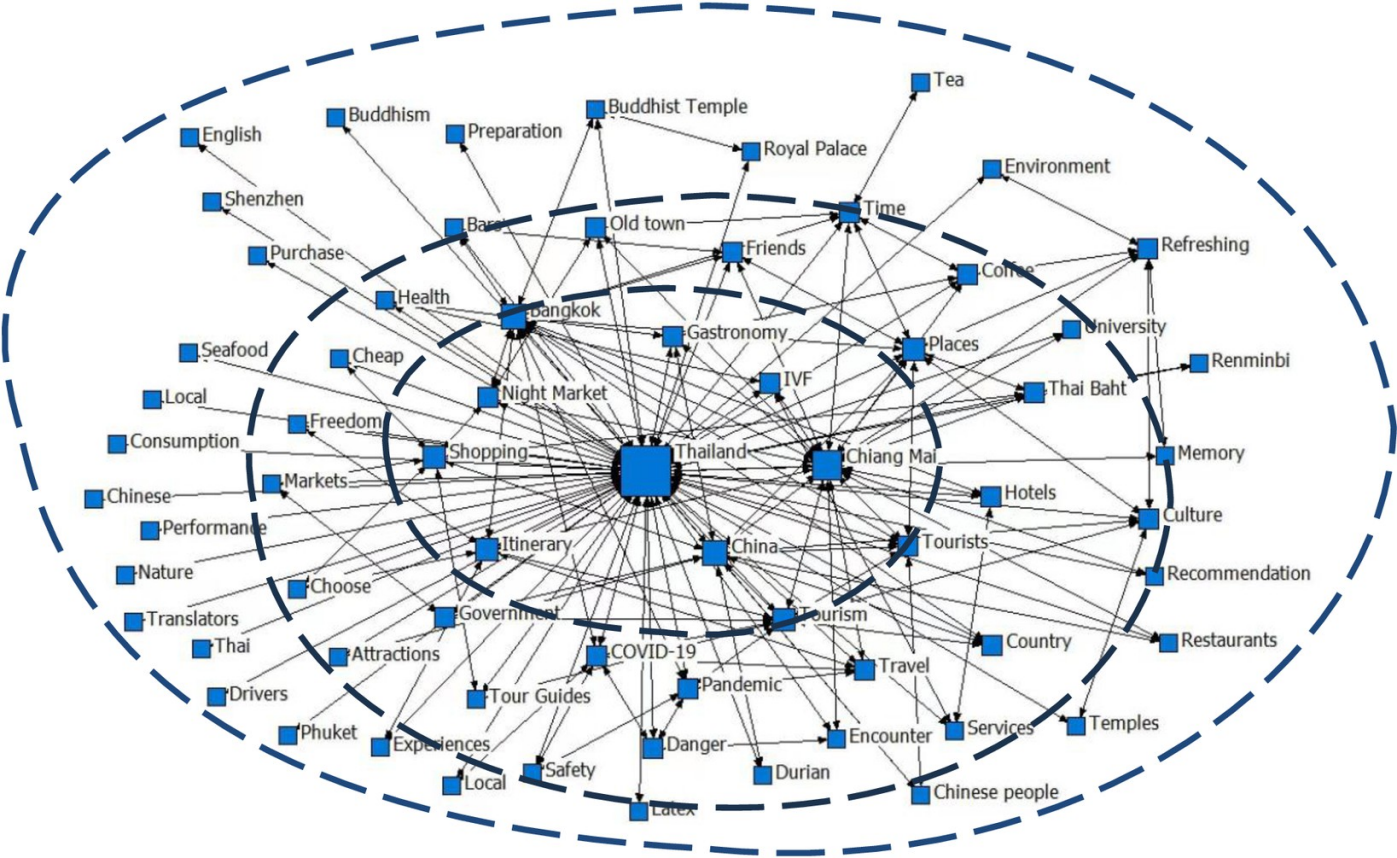
Netography to the perceptive keywords among the Chinese outbound tourists to the destination image of Thailand.

Furthermore, the next stage of this current research has been further analysed through a linkage between these summarized keywords, as shown below in Fig 2. This study methodology clarifies how perceptive recognitions contribute to the construction of overall hierarchy in the society, how structural order of society imposes obligations on each person, and hot this influences behaviours, beliefs, and perceptions [86]. Respected advocates of algorithmic descriptions have written publications on the implementation and general perspectives on these topics [87]. To be more specific, some of these abstracted travelogues would be used to discuss how the (imaginary) destination image of Thailand is constructed, what the projected and perceived personalized tourism destination image of Thailand is, and the concern regarding Chinese tourists’ pursuits of slow tourist consumption, modernity, and individual freedom. These will be examined further in the following subsections of the research discussion section.

The research presents a graphical representation of three concentric rings to illustrate the conclusion that Chinese individuals’ perception of Thailand’s tourism sites may be categorized into three distinct levels. The first core level is the initial phase. Regarding the primary attractions in Thailand, Bangkok and Chiang Mai cities are the most captivating options for Chinese travellers. The most commonly discussed topics in Chinese tourists’ travelogues include Thailand’s cuisine (gastronomy), night markets, shopping opportunities, and vacation itineraries. Surprisingly, one of the keywords showcased in this graph is the medical tourism, which is evident in the discussion of In Vitro Fertilization (IVF) issues in Thailand among the Chinese travelogues. Subsequently, the second level of their concerns are relating to more issues. The keywords in this level can be classified into four distinct categories: shopping experience (Services, Encounter, Travel, Thai Bhat, Markets), travel freedom of choice (Freedom, Choose), attractions and scenic spots (Attractions, Old town, Tour guides), and safety experience (COVID-19, Pandemic, Safety, Danger), the tourists’ place of origin (Shenzhen etc.). Thirdly, some more issues, for instance, language barriers, diverse variety of exotic cultures, and Buddhist culture gained greater significance at the third level. This demonstrates that Thailand remains the foremost and favoured tourist destination in the perception of Chinese tourists. Chinese tourists have consistently favoured Thailand as a popular place for travel. Thailand was selected as their initial outbound target due to its proximity to China, streamlined entrance processes, and thriving local tourism industry. Essentially, they have the freedom to depart promptly upon expressing their intention, with minimal ambiguity and concerns. Ultimately, prioritizing safety takes precedence above enjoyment and relaxation. As such, the findings of this paper may serve as important guidance to the decision-makers and the academia as a reference to analyse / publicize / promote the Thailand Tourism Destination Image in an extensive global scale.

## Discussion

### Before Chinese tourists come: Constructing an imaginary image

The above results exemplified how the Chinese outbound tourists perceive the destination Thailand. The development of the tourist sector is a major strategic objective that must be pursued to improve the image of the nation as a world famous tourism destination, increase the employment rate and improve the soft power that underpins national competitiveness, especially under the guiding strategy of Thai creative tourism [88]. The image of a tourist destination, the appeal of cultural qualities, and the local affiliations are crucial to its success and survival in the face of intense market competition [89,90]. According to the results of the current study, studying the major components of tourist destination image and determining the significant assets of tourism destination image has become a monumental task. The findings of the study above identify the regional specificity of Thai culture. Meanwhile, the construction of a Buddhist temple in the Thai kingdom increases the appeal of Thailand. The level of service given by hotels and restaurants assists travellers in forming an impression of the Kingdom of Thailand. Moreover, the reputation of coffee shop location contributes significantly to the destination’s attraction. Airport accessibility also contributes to image development. Meanwhile, it is crucial for the expansion of Thailand’s tourism industry to create the image of the destination and engage in extensive marketing [91–93]. This study reveals that Thailand has engaged in constant marketing and long-term maintenance of a solid image as a tourist destination, which is one of the most important achievements of the Thai government in promoting its image.

The marketing of the image of the location should be co-created at all stages of the consumer journey, including before, during, and after a tourist’s vacation in the destination [35,94]. Before a Chinese customer decides where to go on the upcoming vacation, the marketer of the destination’s image of Thailand seem to have taken into consideration the following aspects to build up the tourists’ imagination of the destination. The following sections use the outcomes of the data processing to demonstrate the main arguments proposed in the introduction section of the present paper.

First, customers in the tourism industry are quite knowledgeable about reputation, which makes it a suitable quantitative factor for evaluating image assets, as is done in the current study. In the current case of Thailand, while examining the popularity of destination Thailand, the word expressing customer’s favour over destination Thailand has been mentioned 94 times in different contexts in the travelogues of Chinese outbound tourists.

Second, the popularity of an image is a result of the target consumer group’s learning, familiarity and retention of knowledge linked to a tourist location [11,36]. To compete effectively in a market where competition is fierce, a well-known tourist location must have an image that distinguishes it from other destinations on the market. For instance, in the above research results, Thailand, Bangkok, Chiang Mai, have been constantly mentioned by the travelogues. Marketing professionals are responsible for sculpting and polishing the image’s highlights, identifying and establishing the unique characteristics that cannot be replicated by others, and distributing the image through strategies such as overall marketing and event marketing. Reputation is built on popularity since consumers’ perceptions of a location’s value might be correctly reflected by its reputation.

Third, after the image reputation has been created, it is important to consistently promote the image reputation in order to attract tourist consumers to choose one site over others [95,96]. This causes customers to choose one area over other locations. The construction of a tale around a picture paves the way for the production of an atmosphere that is emotionally nourishing, and the actualization of the fundamental ideas contained within the image [97–99]. Such approaches, as shown in the research results, make it possible for Chinese outbound tourists to build a desire to travel to Thailand and explore unknown areas within the territory, and then provide them the opportunity to do so.

### Projecting a customized tourism destination image for Thailand among Chinese outbound tourists

The image of a tourist destination serves two purposes: It allows for the personalization of the emotional experience [100] and it facilitates the customer recognition of the promoted destination image [101–103]. The sole destination image that customers and prospective tourists have of a tourist destination is the impression that consumers have of that place [104]. It is necessary to explore how to incorporate the image of one’s own country into one’s personality, in addition to promoting the image of a tourist destination as a desirable location for vacationers to visit. *Feeling (65)*, *friendship (90)*, *being in favour of (94)*, *lifestyle (64)*, *local specialty (98)*, *experiences (46)*, *and locality* are some of the emotional and personalized keywords that were found in the travelogues written by Chinese tourists for the current case study. These words were used to describe how the tourists felt about their travels in destination Thailand. This stage of destination promotion occurs both before and after the process of marketing the image of a tourist location. To further interpret the essential entailment of these keywords, for instance, *the friendship* (with an occurrence of 90 in this research), government-level relationship can be taken into consideration [105]. The collaboration and diplomatic amity between governments, typified by the relationship between Thailand and China, undoubtedly constitute a significant determinant in shaping Thailand’s destination image. This intergovernmental rapport not only cultivates trust but also facilitates the establishment of agreements aimed at streamlining tourism, exemplified by measures like visa facilitation and fostering interpersonal connectivity. Collaborative marketing initiatives, extensive international exposure, and cultural exchanges further bolster Thailand’s allure on the global stage. Furthermore, the harmonious governmental relationships engender a perception of safety and stimulate economic growth, thereby augmenting the attractiveness of Thailand as a tourist destination [106]. Nevertheless, it is imperative to recognize that the broader composition of the destination image is intricate, influenced by a myriad of factors including the natural environment, cultural richness, safety standards, and the overall tourist experience. Consequently, while government-level amicability is undeniably pivotal, it represents just one facet within the intricate tapestry that shapes Thailand’s tourism image. A holistic and comprehensive approach is thus indispensable for optimizing the appeal of Thailand to international tourists.

As a consequence of the political instability in 2008–2009 caused by the confrontation on the streets, the number of international tourists visiting Thailand in 2009 decreased by 16% compared to the previous year [107]. The political unrest in May 2014 also impacted the desire of international tourists to visit Thailand, resulting in a decrease in the number of international tourists that year [108–110]. The Thailand Tourist Authority (TAT) unveiled a new integrated international tourism image marketing campaign in 2015, adding Discover Thainess as Thailand’s essential value to the country’s current national tourism slogan [111,112]. In his 2014 message, Prime Minister Prayut Chan-o-cha asked the Thai people to preserve the country’s 12 Core Values and to be more friendly and welcoming to foreign tourists [113–115]. The Thai government designated 2015 as the Year of Tourism of Thailand, and the Tourism Authority of Thailand (TAT) aggressively promoted the global tourism marketing campaign ‘Discover Thainess’ during the same year. As a direct consequence of this massive marketing effort, the number of tourists who travelled to Thailand increased and recovered. These activities demonstrated the efforts that the Thai government has been doing to build and construct an ambivalent destination image by means of a variety of engaging and varied endeavours.

### A perceived destination image of Thailand during the visit

During the visit of Chinese outbound tourists, the image of the tourist destination should focus on creating comfortable infrastructure [116], convenient transportation [117], friendly tourist attractions [118,119], creating a good tourism hardware and technological environment [120–121], and providing high-quality tourism service experience to improve the quality of tourism services [12]. These demands have been shown to be represented in the study findings, as Chinese tourists who travel outside the country exhibit a significant interest in *friendship (90)*, *restaurants (72)*, *breakfasts (70)*, *durian (67) and the quality of the life*. These results have led to the conclusion that establishing soft tourism supporting services is crucial to improve the reputation of a tourist destination.

Tourism destination image marketing should focus on fostering image loyalty [122–124]. According to the findings of the present investigation, the Thai government has been effective in attracting Chinese outbound tourists to visit Thailand multiple times, developing a preferred aptitude, and developing strong emotional ties [56,125–127]. According to the findings of this research project, it is possible to obtain a considerable premium revenue impact by anticipating that tourists would return to a destination a second, third, or even a significant number of times, and even pay higher costs for the privilege.

The acceleration of the rate at which tourist sites are being built has resulted in a growing tendency towards the standardization of the products and amenities provided in tourism, as well as the shaping of tourism’s overall image. These changes have been influenced by the positioning of destination image. In the perceptions of the Chinese outbound tourists, they communicated their concerns to Thailand’s social environment political-economic atmosphere, expressing the sentiments of the place and their intention to demystify the destination in curiosity. When the average income of tourists is higher and the tourism sector is more established, there is a larger need for businesses in the tourism industry to provide services that are tailored to the specific needs of tourists. For instance, according to the current study, there are extensive discussions about the de-mystifying the destination Thailand while the tourists are talking about *Temples (72)*, *Cultures (30)*, *Thai people (69)*, *Kingdom (44)*, *Atmosphere of Lady Boys (30)*, *Buddhism Amulets (53)*, and so forth. These concerns have established a goal for the government agencies that are responsible for the promotion of the destination’s image, and that goal is to create an appealing tourism image that includes benefits, emotions, and opportunities for self-actualization, as well as to establish a core tourism image that promotes the value of emotional experiences.

### Chinese tourists’ pursuits for slow tourist consumption, modernity, and individual freedom

To understand the perceptions of Chinese outbound tourists of Thailand as a tourist destination, it is suggested to view travel experiences from both the demand side with tourists’ consumption in the destination and the supply side with products designed in the destination. First, the consumption side has changed with new forms of traveling mode. With the rapid growth of the economy in China in recent decades, the Chinese government and the Chinese people started to realize the importance of ecology, quality-oriented lifestyles, and increased awareness of slow tourism as a form of quality life. This is exemplified by Li’s judgement [128] that Chinese’s outbound tourism is now changing from 1.0 version to a 2.0 version. Bao et al. [129] also proposed a third wave for Chinese outbound tourism development while profiling the middle-aged Chinese outbound elite tourists. In this regard, tourism with the genre of modernity has seen its emergence as a popular academic issue and in the social media context [130]. Slow tourism, an example for slow and soft mobilities, has been associated with environmentally friendly tourism, sustainability with longer-stay travel [131–134]. To the new generation of Chinese outbound tourists, slow tourism encompasses many experiential dimensions: locality involvement with food and beverage, as can refer to the frequency of *hotels (132)restaurants(80) food(17)*, *tea (51)*, *durian (67)*, slow-paced travel in association with local patrimony and cultural diversity while mentioning *lifestyle (64)*, *the opportunity to choose (59) and slow-paced life (29)*, as well as an amiable encounter with local communities, while they constantly mention f*riendship (90)*, *as well as Thai locals (69)*. These characteristics of slow tourism were disclosed in detail by the netography that was discussed previously, considerably strengthening the conceptual connotation of the expansion of Chinese tourists traveling outside the country. Slow tourism is becoming more popular among Chinese tourists of the younger generation as an alternative to the prevalent concept of living a fast-paced lifestyle to support mass production in the modern day [135,136]. Economic development calls for fast-paced production, as Fordism has shown, “globalized homogenisation, prioritized globalization, standardization and rationality” [132, p2]. Ritzer [137] analysed the culture of the current era for seeking for rationalized, efficient, standard, and predictable commodity production, and notes that this fast-paced mass production might not be beneficial to the overall social wellbeing. This is particularly true in the tourist and hospitality business in this new period and new environment, according to the authors’ reasonable judgment since tourism is geared at improving general societal well-being. According to the findings of this study, a younger generation of Chinese tourists has shown increasing sensitivity to the fundamental aspects of excellent tourism by making many observations in their travelogues. Chinese travellers who have travelled outside the country have gained a new understanding of the fundamental components that make up the essence of slow tourism. Not only is it the antithesis of fast-paced tourism and the conventional idea of all-inclusive vacation packages, but it also integrates an awareness of ecological problems and a dedication to the development of sustainable tourism.

## Conclusions

To achieve greater levels of success, the government organizations that are responsible for marketing tourist destinations need to conduct research and monitor the numerous ways in which tourists perceive the locations that they advertise, and then adjust their branding tactics appropriately. Content analysis and Netography were used on four of the most popular blogs and websites to share tourist experience as part of this research project. The results of these analyzes led to the discovery of Thailand as a digitally active and emotionally positive destination for Chinese mainland outbound tourists. The research findings reveal interesting insights that have significant implications for destination branding in terms of promotion. These insights are derived from the analysis and evaluation of widely used expressions in user-generated content across various blogs and websites.

This investigation dispels the myths that Chinese tourists have about Thailand and contributes to understanding the construction of Thailand’s image as one of the world’s most popular tourist destinations. This new generation of Chinese travelers who travel outside of China has self-positioning mentalities and development tendencies that put a premium on sensation-seeking, technology facilitation, novelty, and demystifying the region they are visiting. These traits point to a feeling of modernity among the connections that Chinese tourists traveling outside of China have with Thailand. To obtain a depth sense of Thailand’s tourism image construction, the current research adopted a qualitative methodology to analyze the depictions of Thailand’s tourism image construction in four significant user-generated travelogues. This research was intended to provide perspectives on inbound tourism phenomena in Thailand by analyzing media stories. As this area is mostly disregarded, it is anticipated that the present study will serve as foundation research that will promote further study into the subject in Thailand and beyond.

### Contributions

This article significantly contributes to the existing literature on destination image in several ways. Firstly, it underscores the imperative for destination marketers to diligently monitor and assess the online portrayal of a destination’s image, ensuring its alignment with the intended perception. This insight equips marketers with the strategic acumen necessary for addressing unfavourable perceptions and bolstering positive ones within the digital domain. Secondly, the article demonstrates a pioneering approach by seamlessly integrating netography and text-analysis methodologies, offering genuine and nuanced insights into the perception of Thailand’s destination image among Chinese outbound tourists. This innovative approach not only enriches the discourse but also aids in fostering continuous enhancement and development of the destination’s image. Thirdly, it illuminates the perspectives of the burgeoning community of Chinese travellers venturing beyond their homeland to explore Thailand, an internationally acclaimed premier tourist destination. This facet not only augments our understanding of evolving tourism dynamics but also informs strategic decision-making for stakeholders in the industry. Furthermore, the article proffers forward-thinking recommendations, aimed at guiding future research endeavours and deepening our comprehension of destination image reconstruction. Lastly, within the context of the profound ramifications of the COVID-19 pandemic on global tourism, particularly within Thailand, this research equips industry stakeholders, including business proprietors and destination marketing sectors, with invaluable insights to enhance their competitive advantage amid these challenging circumstances.

### Limitations and future research agenda

It is essential to consider the limitations of the current study. First, a thorough comprehension of the text needs a mix of semantic interpretations, even if calculating the frequency of a word’s use might give some information. For instance, the term “*friendship” (of 90 occurrences)* emerges as one of the most frequently used keywords in Chinese tourist travelogues, ranking 11th among the list of significant terms cited in travelogues (see Fig 2 above). However, the term’s usage could encompass various contexts, such as encounters with Thai friends, the fostering of China-Thai friendships, or camaraderie within tour groups, or potentially encompass all these concurrently. It is strongly recommended that future research endeavours incorporate contextual subject analysis into their content mining algorithms for keyword and topic identification, thus facilitating more precise outcomes.

Secondly, owing to limitations in resources and budget constraints, the Chinese-character documents were not translated into English prior to the text processing phase of this research. Had the Chinese travelogues been translated into English for this study, the results may have exhibited some degree of variation and potentially offered greater insights. Future research could explore the implementation of machine translation techniques within the text processing framework to enhance accuracy and mitigate research expenses. Future research might include conducting further evaluations in several languages or additional studies under different research conditions to better understand a broader range of perspectives on Thailand as a tourist destination. The data might assist the Thai administration in developing a more targeted marketing plan that addresses different consumer groups.

Finally, prior to the commencement of the 11th Golden Week for Chinese tourists in October 2023, Thailand’s general election concluded, resulting in the establishment of Sathiratha as the Prime Minister of Thailand. Subsequently, the government initiated the implementation of several new policies aimed at bolstering the country’s tourism industry and rectifying its international reputation as a preferred tourist destination. Initially, it is noteworthy to mention that a considerable quantity of apprehensions has been made with regards to syndicates involved in telecommunications fraud in the northern region of Myanmar. China, Thailand, Myanmar, and Laos are engaged in collaborative efforts to combat telecommunication fraud syndicates, thereby establishing conducive external circumstances for Thailand to restore its positive reputation as a globally recognized tourism destination. Furthermore, Thai embassies located overseas have formally declared the implementation of a policy that allows Chinese tourists to enter the country without the requirement of a visa. To enhance the tourism sector in Thailand, foster cultural interactions, and strengthen the bond between the Chinese and Thai populations, the facilitation of entrance for Chinese tourists into Thailand is scheduled to commence at the onset of the tourist season in late September 2023. Chinese tourists are exempt from the obligation to get visas for entry into Thailand. Additionally, this strategy is expected to enhance the tourism sector in Thailand, thereby playing a crucial role in encouraging economic growth. In addition, the Prime Minister of Thailand made a formal declaration of hostilities against illicit substances, placing particular emphasis on the reintroduction of marijuana for medicinal purposes. Chinese tourists who choose to return to Thailand during the peak seasons would be experience optimal levels of safety and convenience under the joint TDI policies [138,139]. Thus, all these recent efficient steps undertaken by Thailand to enhance the tourism economy have garnered widespread approval from various segments of society and worthy of further academic observation and research.
